# Navigating regulatory and analytical challenges in live biotherapeutic product development and manufacturing

**DOI:** 10.3389/frmbi.2024.1441290

**Published:** 2024-08-15

**Authors:** Microbiome Therapeutics Innovation Group, Dana Barberio

**Affiliations:** ^1^ Washington, DC, United States; ^2^ Edge Bioscience Communications, Sherborn, MA, United States

**Keywords:** live biotherapeutic product, microbiome therapeutics, regulatory, analytical testing, manufacturing, drug development, potency, bioburden

## Abstract

The recent FDA approvals of Rebyota™ and Vowst™ represent landmark milestones within the burgeoning field of live microbiota-based products. Future microbiota-based treatment approaches also hold significant promise for treating patients with a myriad of diseases and disorders, yet substantial hurdles hinder their development and utilization. Foremost, existing regulatory frameworks governing live biotherapeutic product (LBP) manufacturing development have notable gaps, requiring comprehensive expansion and refinement. Along with regulatory challenges, hurdles remain in the optimization and validation of analytical methodologies essential for characterizing LBPs, including for microbial identification, potency, and bioburden. To address these challenges, Microbiome Therapeutics Innovation Group (MTIG) spearheaded collaborative efforts, engaging industry leaders and the FDA in discussions aimed at catalyzing improvements in LBP analytics and refining the current regulatory landscape. Extrapolating on feedback from these discussions, this review highlights challenges and identifies critical gaps. Specific recommendations for future regulatory guidance are proposed, as are recommendations for interactions that developers can take now with regulatory agencies to support the development of maturing guidance. Key analytical factors to consider in LBP development are reviewed, highlighting strengths and weaknesses of various methodologies. Collaboration among regulatory and government agencies, industry, and academia, facilitated by coalitions like MTIG, will be instrumental in ushering the microbiota-based therapeutics field into the next phase of approvals and advancements, ultimately benefiting patients.

## 1 Introduction

As scientists unravel the causal relationships between commensal microbial communities and human health, opportunities for innovative therapies continue to expand. Early interventions were focused on fecal microbiota transplants (FMT), but the modalities investigated have expanded to include prebiotics, probiotics, postbiotics, donor-derived microbiota-based biotherapeutic products, and defined single/consortium live biotherapeutic products (LBPs) (**Table 1**). These modalities vary widely in their regulatory frameworks. For example, probiotics are considered foods/supplements, lack a commonly accepted definition among regulatory agencies (**Table 1**), have not been approved as drugs by the FDA, and require an Investigational New Drug (IND) application filed if used in a clinical setting to treat, prevent, or cure disease (Food & Drug Administration, 2006, 2016, 2018, 2023a; World Health Organization, 2006). Probiotics used “off label” and without an IND have posed safety hazards in some cases, with the FDA issuing warning statements (Food & Drug Administration, 2023a). In 2013 the FDA ruled that FMT would be regulated via the IND pathway (Merrick et al., 2020). In broad strokes, the 2013 ruling provided a pathway to therapeutic approval for live microbiota therapeutics, and two have now been approved: Ferring’s Rebyota™ and Seres Therapeutics’ Vowst™, representing landmark milestones for the field (Food & Drug Administration, 2023b). Both are donor-derived microbiota-based biotherapeutic products designed to address dysbiosis within the gut microbiome for the prevention of recurrent *Clostridioides difficile* (rCDI) infections, and both are a significant advancement beyond FMT in that they have controlled manufacturing processes, defined analytical testing methods, and established clinical performance (Lavoie et al., 2023).

**Table 1:** Nomenclature.

Term	Definition
**Live biotherapeutic product (LBP)**	A biological product that 1. contains live microorganisms, such as bacteria, viruses or yeast; 2. is applicable to the prevention, treatment, or cure of a disease or condition of human beings; and 3. is not a vaccine (Food & Drug Administration, 2016)
**Donor-derived microbiota-based biotherapeutic product**	Microbiota-based product that is derived during the manufacturing process from donor materials (e.g. stool samples) as the source for the formulated microorganisms
**Defined consortia live biotherapeutic product**	Fermented live biotherapeutic product that is derived from multiple cultivated naturally occurring microorganisms of defined, standardized composition (Ducarmon et al., 2021). Alternatively termed “designed” consortia live biotherapeutic product (McChalicher and Aunins, 2022)
**Defined single live biotherapeutic product**	Fermented live biotherapeutic product that is derived from a single cultivated naturally occurring microorganism as the source for the formulated product. Alternatively termed “designed” single live biotherapeutic product (McChalicher and Aunins, 2022)
**Probiotics ^1^**	Whole, live microorganisms that are ingested with the intention of providing a health benefit (such as supporting digestion and nutrient adsorption in the intestine) (Food & Drug Administration, 2006) OR Live microorganisms, which when administered in adequate amounts, confer a health benefit to the host (World Health Organization, 2006) OR Whole, live bacterial strains or other microorganisms intended for non-therapeutic health benefits, commonly used in the US as dietary supplements, and as such not currently subject to FDA approval (Microbiome Therapeutics Innovation Group, 2024)

^1.^Probiotics lack a commonly accepted definition, both generally and among regulatory agencies.

Despite the promise of these approvals, substantial challenges persist for development of additional microbiota-based modalities, particularly with respect to understanding regulatory guidance and expectations. For instance, in 2016 all live microbiota-containing therapeutic modalities, including those now approved, appeared to fall within the FDA’s LBP classification, whereas there are now indications that this classification may be limited to modalities whose manufacturing starts from laboratory-defined microbial strains (Food & Drug Administration, 2016). Since no such products are yet approved, this review will focus on unresolved questions associated with the analysis and regulation of these laboratory-defined single or consortia LBPs. To date, FDA’s key guidance for LBPs is limited to only one document, issued in 2016 (Food & Drug Administration, 2016). While the lack of subsequent guidelines or further standardization has enabled continued innovation, it can also lead to unclear expectations and differing baseline assumptions which may delay clinical availability of new drugs. These gaps motivate diligent communication between LBP developers and regulators and demand further expansion and refinement of guidance as the field matures.

Throughout development and manufacturing of defined single or consortia LBPs, characterization of purity, potency, and identity is critical for patient outcomes and safety, as well as the quality documentation required for regulator assessment. Unfortunately, the selection and validation of analytical assays remains challenging. Likewise, health authority expectations and the applicability of existing Good Manufacturing Practice (GMP) guidance for manufacturing of these products is incomplete and often not harmonized across regions. To address these and other issues, Microbiome Therapeutics Innovation Group (MTIG) hosted a workshop at the 2023 Microbiome Connect conference to engage industry leaders and the FDA. Here we will expound upon these discussions to provide industry views on opportunities to improve analytics of LBPs, motivate foundational research across academia and industry, highlight current regulatory gaps, and propose specific interactions between regulators and developers to collaborate on maturing guidance in the field.

## 2 General needs and gaps in analytical testing

Analytical testing for the comprehensive characterization and release testing of any LBP encompasses key parameters such as identity, potency, purity (including microbial bioburden and contamination control), and stability (Food & Drug Administration, 2016). Definitions for these parameters have been recommended by the FDA, but specific analytical methods are not standardized. When developing such methods, it is imperative to ensure precision, accuracy, selectivity, specificity, and operational robustness in quality control processes to demonstrate lot-to-lot product consistency.

**Figure 1:**
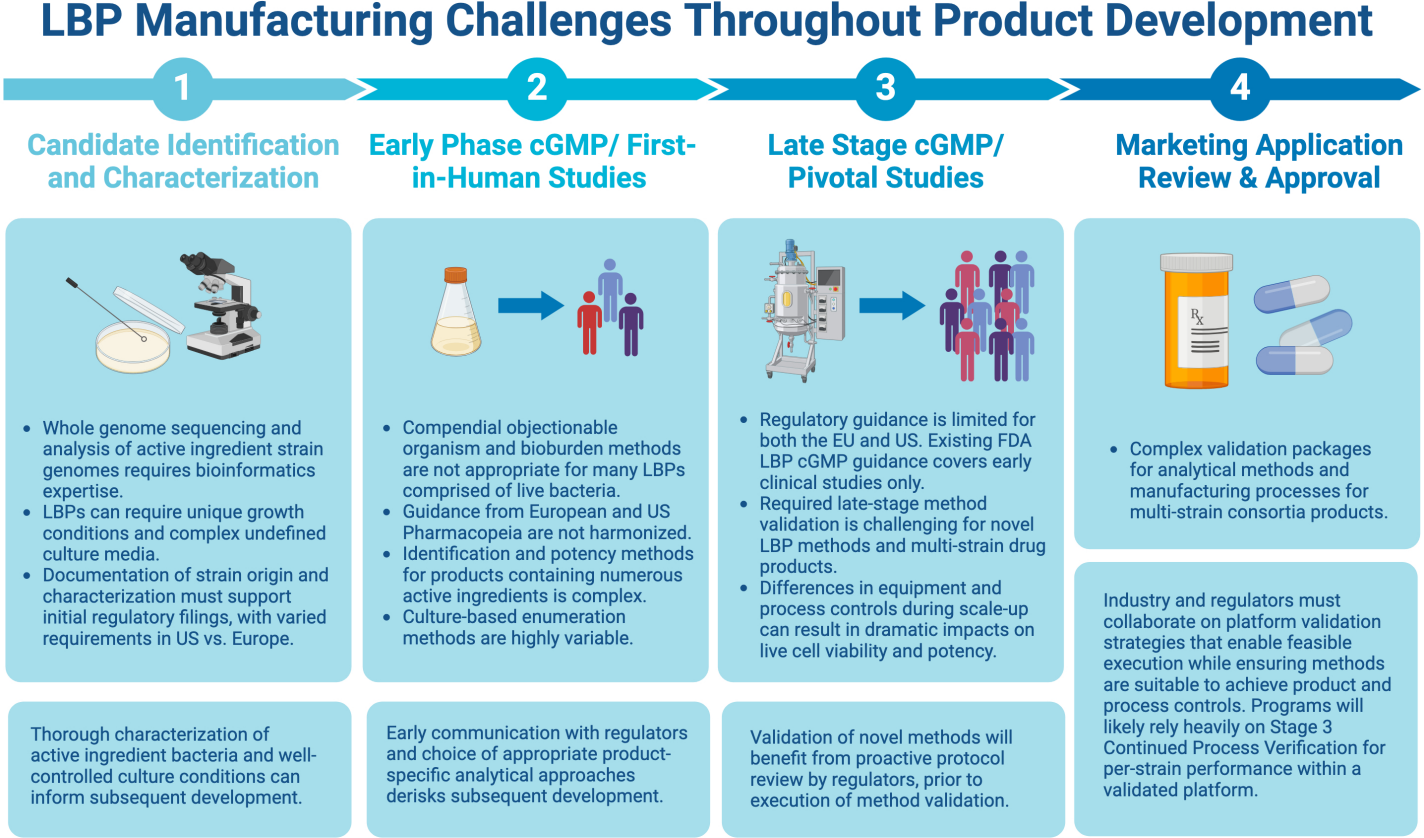
Challenges and potential solutions at each step of the product development cycle. Figure created with BioRender.com.

Assays traditionally used for biologics, including compendial methods, may perform poorly with respect to the above attributes when applied to LBPs, stemming from the complexity of the starting raw materials and various additional factors, such as biological diversity, number of strains, strain-to-strain interference, etc. Because of this, assay optimization and acceptance criteria for qualification and validation must be well-justified for each specific LBP, route of administration, and target population. The complexity and novelty of these assays may result in high costs for development and implementation, especially during early-phase development. Consistent execution will rely upon well-trained QC technicians and an emphasis on retaining staff with specific expertise. A balance of innovation, cost considerations, and regulatory standardization will be necessary, understanding that all methodologies will require a rigorous level of validation to support licensure and marketing authorization. Therefore, as the field continues to mature, successful end-to-end development requires mechanisms for active collaboration with regulators and within the industry in excess of the typical phased interactions to enable meaningful interpretation of clinical results and validation performance for both test methods and manufacturing processes. An overview of the challenges and potential solutions at each step of the product development cycle, from LBP identification/characterization through the marketing approval stage is presented in **Figure 1** and discussed in detail below.

## 3 Evolving microbial identification methods

Traditional methods of microbial identification (ID) testing are based on cell morphology, colony morphology, and metabolic phenotypes, and are often insufficient to characterize LBPs, especially some multi-strain products displaying overlapping phenotypes or diverse growth requirements. Alternative ID methodologies include 16S rRNA gene sequencing, taxon-specific quantitative PCR [qPCR], MALDI-TOF mass spectrometry, and other biochemical or functional assays (Vandeputte et al., 2017; Galazzo et al., 2020; Jian et al., 2020; Paquet et al., 2021; Coryell et al., 2023). Some of these analytical assays are more easily validated than others based on previous use for established modalities, thus drug developers must assess overall suitability for the proposed use, including performance criteria (e.g., sensitivity and selectivity), implementability, and operational robustness. For multi-strain LBP release testing, ID methods may overlap with expectations for per-strain quantification to support potency.

There are a variety of factors to consider while selecting the optimal ID testing method, including accuracy, sensitivity, potential biases, and cost-effectiveness (Vandeputte et al., 2017; Galazzo et al., 2020; Jian et al., 2020; Coryell et al., 2023). The requirement for additional expertise and the higher cost of alternative methods such as MALDI-TOF can be a barrier for sponsor companies. Developers may consider combining methods to provide a more comprehensive characterization. For example, to overcome ID and enumeration challenges associated with LBPs, MALDI-TOF has been combined with colony forming unit (CFU) enumeration to simultaneously identify and enumerate viable active ingredient bacteria (Coryell et al., 2023).

With LBPs, product-specific ID is required by the FDA and European Medicines Agency (EMA), and the FDA recommends using at least two complementary methods for both ID and active ingredient assessments (Paquet et al., 2021). For example, a qPCR assay may be used to delineate strains with identical 16S rRNA gene sequences. Overall, using an ID method that can ensure a qualitative yes/no on batches produced over time is desirable.

To ensure assays have adequate sensitivity and specificity, benchmarking standards/controls are important throughout the development and manufacturing process. Standards are useful for any molecular-based assay used: 16S rRNA sequencing, MALDI-TOF, or the more exploratory option for LBPs, metagenomics sequencing. The use of appropriate sequencing controls is critical, including internal “spike-in” standards to address any variability in assay methods performance (Tourlousse et al., 2017). Nevertheless, the route remains unclear for validating sequencing as an approval-enabling product specification and further evaluation is needed to assess the suitability of any method for routine product release testing in the context of a commercial LBP.

## 4 Challenges in potency testing

Throughout the manufacturing process, including upstream fermentation, final release testing, and long-term storage, monitoring of LBPs for viability is imperative. Stabilization may be a challenge with certain formulation techniques and compositions, and viability of actives may be impacted when not under ideal conditions. Many LBPs are developed using a viable cell specification for potency release testing of drug substance and drug product, including monitoring of potency during stability studies. As more LBPs advance through late clinical and commercial development, an increase in understanding of their mechanism of action may facilitate identification of other functions or characteristics that are critical to clinical efficacy and that can be deemed gold-standard measures of potency (Paquet et al., 2021)

All methods of viable cell enumeration via CFU testing of active ingredient strains generally need to be tailored to the LBP. The different strains present in multi-strain LBPs may have unique growth requirements, diverse colony morphologies, or strain-to-strain interferences which can affect method performance. Further, common culturing methods and media may not even be feasible or amenable to validation for some strains. To address some of the challenges and shortcomings of traditional enumeration methods, alternative methodologies for LBP potency testing can be considered, including propidium monoazide (PMA) viability qPCR, flow cytometry quantification and sorting, impedance-based methods, or other biochemical or functional assays (Kobayashi et al., 2009; Reyneke et al., 2017; Galazzo et al., 2020; Chen et al., 2024). All of these alternative methodologies also have technical challenges/shortcomings in their application for potency testing but are valuable to explore.

Numerous factors must be carefully evaluated in establishing potency testing. In addition to release criterion, potency is a key metric of long-term stability and consistency between drug substance and drug product. Further, performance of potency test methods and product critical quality attributes should be considered when establishing specifications and testing strategies for dosage unit uniformity (U.S. Pharmacopeial Convention, 2011).

## 5 Bioburden and contamination control strategies

Monitoring microbiological impurities in LBPs is especially important as LBP manufacturing methods often include growth-promotion operations for fastidious organisms and frequently exclude operations for inactivation or clearance of non-product organisms. Guidance for acceptable levels of bioburden and specific microorganisms of concern is available for drugs in general but is incomplete for LBPs (Food & Drug Administration, 2016; U.S. Pharmacopeial Convention 2016b; Paquet et al., 2021). Compendial test methods, while recommended in FDA guidance for early clinical trials with LBPs, have not been developed for LBPs. Consequently, their potential lack of specificity may lead to challenges in accurately enumerating bioburden, especially when product-strain breakthrough occurs and confounds the results (Food & Drug Administration, 2016; U.S. Pharmacopeial Convention, 2016a). The European Pharmacopoeia does include LBP-specific chapters related to bioburden and specified microorganism analysis, however, these are not harmonized (European Pharmacopoeia, 2011; Franciosa et al., 2023). Compendial methods for anaerobic bioburden testing are not yet established, and not currently recommended in FDA guidance, though sponsors may need to consider incorporating such testing based on risk assessment of manufacturing operations, facility performance, product characteristics, route of administration, and the proposed patient population (McChalicher and Aunins, 2022).

A risk-based approach should be taken to application of USP <1111>, which provides criteria for existing bioburden limits with specified microorganisms based on sample type and route of administration (U.S. Pharmacopeial Convention, 2016b). Some LBPs warrant tighter limits than those traditionally applied based on route of administration and dosage form. In assessment of risk, challenges inherent to LBP production processes, analytical limitations, and the characteristics of the target population should be considered. Supplemental methodologies for bioburden measurements, such as nucleic acid amplification techniques, may enhance detectability of strains for functionalities which present as a high risk for the intended target product profile (McChalicher and Aunins, 2022).

In addition to bioburden levels, regulatory agencies seek data from developers on the risk of transferability of antibiotic resistance to other bacteria and the risk of causing infection, both of which may be impacted by the levels of contamination, and thus would be part of any safety documentation (Paquet et al., 2021).

## 6 Additional gaps and heterogeneity in the evolving global regulatory framework

Regulatory oversight from the EMA in the EU and the FDA in the US, along with the pertinent ICH guidelines, govern the manufacturing of LBPs (Cordaillat-Simmons et al., 2020). In the US, production of investigational new drugs and biological products are subject to current GMP required under section 501(a)(2F)(B) of the FD&C Act and the IND regulations at 21 CFR Part 312, but the only LBP-specific guidance is a 2016 treatise on Chemistry, Manufacturing and Control (CMC) in early clinical trials (Food & Drug Administration, 2016). No updated guidance is available that considers the field evolution since then, and there remains no guidance for later stage or commercial CMC.

As in the US, there remains a guidance gap in the EU. The EMA coordinates with member states of the European Economic Area and the European Commission to provide regulatory guidelines governing medicinal products (Pharmabiotic Research Institute, 2022). Volume 4 of EudraLex contains guidance on cGMP for medicinal products for human use, and provides principles and guidelines for ensuring the quality, safety, and efficacy of medicinal products during their manufacturing process (European Commission, 2024). Since it does not include a specific definition for LBPs, the applicability of EudraLex Volume 4, e.g., Annex 1 and 2, and Part II remains unclear. Indeed, the only European regulations specific for LBPs are provided by European Pharmacopoeia (Ph.Eur.), via the general monograph 3053 on LBPs for human use, providing rather high-level dispositions on manufacturing and testing, and the two analytical monographs 2.6.36 and 2.6.38 on microbiological examination of LBPs regarding enumeration of contaminants and tests for specified microorganisms, respectively (Franciosa et al., 2023).

These gaps in US and EU guidance leave critical topics unaddressed, such as bioburden and cross contamination control (as discussed), clean room classification, biocontainment, and suitable controls regarding batch-to-batch and product-to-product facility changeover. One critical unaddressed challenge centers on defining the limits for bioburden detection, as this could impact either patient safety or efficacy. More data in that area will drive the type of standards and classifications the industry needs for bioburden controls. Additional shortcomings include guidance on controls pertinent to multi-product/multi-strain facilities and validation methods for platform methods or processes, as well as products containing spore-forming microorganisms (Food & Drug Administration, 2016; European Commission, 2024). A final gap centers on process control and qualification of material suppliers, including medium components often new to cGMP, and the validation status of computerized systems that haven’t been used previously for GMP manufacturing.

The FDA is currently taking an open approach, not providing general guidance for LBPs beyond that for early phase clinical studies, in this newly evolving field. A strength of this approach is that it allows for continued innovation, yet until further guidance is released some ambiguity will remain. To that end, agencies in general need validated assays, relevant data and scientific rationale from LBP developers before solidifying regulation.

## 7 Avenues that developers can pursue now to navigate regulatory requirements

In the short term, developers will need to closely communicate with regulators and propose modifications to established drug development guidance as needed. Bilateral discussions with regulators on all critical steps of their product development should start early in the drug development lifecycle (Charbonneau et al., 2020; Cordaillat-Simmons et al., 2020). This will reduce the risk of a hold for an IND or other delays in advancing promising drug candidates through all phases of development.

It is likely LBP regulations will follow the patterns observed recently for other advanced therapies. For example, gene therapy and cell therapy faced gaps in applicable regulatory guidance and received special and specific regulation by the FDA in part due to their novelty and complexity, and potential safety risks (Eisenman, 2019; Beetler et al., 2023; U.S. Pharmacopeia, 2024a, 2024b). The FDA collaborated with experts, industry, and academia to develop guidelines specific for these emerging fields after clinical success and demonstration of market acceptance (Eisenman, 2019; Beetler et al., 2023).

Likewise, the novelty and complexity of LBPs will ultimately warrant special and specific regulation. By fostering dialogue and knowledge-sharing among industrial, academic, and regulatory stakeholders now, evidence-based LBP regulations may be developed. It will be up to the industry to push this consensus-building, just as they did with gene therapy.

## 8 Recommendations for longer-term development of regulatory guidelines

To move toward more specific regulatory guidance, FDA’s guidance on “Early Clinical Trials with LBPs: CMC Information” should be expanded to cover missing topics particular to LBPs, including bioburden and objectionable organisms, multi-product/multi-strain facilities, LBP-specific technical handling steps, and other topics as discussed here (Food & Drug Administration, 2016). In addition to addressing these gaps, regulations for later stages, including validation strategies for platform test methods and production processes, may be developed from existing drug development regulations, such as 21CFR Part 600 for biologics (Code of Federal Regulations, 2014), 21 CFR Part 210 for cGMP for manufacturing drugs, and 21 CFR Part 211 for cGMP for finished pharmaceuticals (Code of Federal Regulations, 2024a; b) Regulators can draw on the lessons learned from approval of Rebyota™ and Vowst™. Programs such as FDA’s Breakthrough Therapy designation and Priority Review are intended to provide accelerated pathways to development and approval of therapies meeting the associated criteria, as was the case for both Rebyota™ and Vowst™. Clear, consistent, and timely updates by regulators as expectations for LBPs evolve ensure sponsors can achieve the intended benefits of these programs.

## 9 Discussion

Improving analytical technology for LBPs and continued collaborations for strategic implementation are critical for advancing this field. Facilitating these collaborations are organizations such as MTIG, an acknowledged liaison to the FDA, and Pharmabiotic Research Institute (PRI), Europe’s Microbiome Regulatory Science Expertise Center, both of which support the regulatory development of microbiome therapeutics by fostering communication among regulatory agencies, industry experts, and other stakeholders. Contributions by USP, National Institute of Standards and Technology (NIST), and National Institute for Innovation in Manufacturing Biopharmaceuticals (NIIMBL) can also move the field forward. Workshops, conferences, publications, and white papers are highly useful for facilitating and capturing the value of such collaborative projects.

Highlighting the benefit of these collaborations, the roundtable workshop led by MTIG inspired some solutions, presented here, in the ongoing effort to address challenges and future directions. Here we proposed specific interactions that developers and regulators can consider to collaborate on maturing guidance. For example, developers should communicate with regulators on all critical steps of their product development, early and often in the development life cycle, even proposing modifications to established drug development guidance as needed. In general, regulatory agencies need LBP developers to provide relevant data, scientific rationale, and qualified or validated assays (depending on the phase of development) before solidifying regulation (Parenteral Drug Association, 2012). Developers can thus support guideline development with these actions. By addressing the challenges and shortcomings of analytical methods used in LBP ID, potency, and bioburden testing, we proposed technical strategies and corresponding regulatory factors for developers to consider.

There is also a proactive role available to the FDA, EMA, and other regulatory bodies, as well as organizations such as MTIG, PRI, and the European Microbiome Innovation for Health (EMIH), to promote understanding of regulatory requirements via workshops and/or educational training programs. For instance, a working group to explore and develop regulatory guidance for the end-to-end process from cell banking to formulation and then finished product management would be beneficial. Another key to advancing these regulatory concepts will be established method standards, as are being championed by NIST (Olson et al., 2015; Forry et al., 2024).

To move the field forward, with LBPs in the pipeline, there is a clear need for more specific regulations, not only for commercial products but across the various stages of clinical development, from early clinical to Phase 3 process validation and commercialization. Most likely there will not be a one-size-fits-all solution since each LBP has unique properties. Nevertheless, there are likely enough commonalities in different LBP modalities to work toward consensus for some aspects. In order to achieve this goal of more defined LBP regulations, collaboration and active engagement between the FDA, EMA, industry, academia, and other relevant governmental agencies is essential. Data sharing and collaboration among developers and researchers as they relate to establishing some consensus in regulatory guidelines are strongly encouraged, as this would accelerate knowledge accumulation and facilitate evidence-based decision-making. Analytical improvements and regulatory solutions are the keys to success in this dynamic field. Novel solutions and a spirit of collaboration will carry this field forward into the next phase of LBP approvals.
